# Association of Household and Community Socioeconomic Position and Urbanicity with Underweight and Overweight among Women in Pakistan

**DOI:** 10.1371/journal.pone.0122314

**Published:** 2015-04-02

**Authors:** Naveed Zafar Janjua, Bushra Mahmood, Junaid A. Bhatti, M. Imran Khan

**Affiliations:** 1 British Columbia Centre for Disease Control, Vancouver, BC, Canada; 2 School of Population and Public Health, University of British Columbia, Vancouver, BC, Canada; 3 Sunnybrook Research Institute, Sunnybrook Health Sciences Centre, Toronto, ON, Canada; 4 Department of Surgery, University of Toronto, Toronto, ON, Canada; 5 Institute for Clinical Evaluative Sciences, Toronto, ON, Canada; 6 Sabin Vaccine Institute, Washington, DC, United States of America; Dasman Diabetes Institute, KUWAIT

## Abstract

**Background:**

Similar to other developing countries, Pakistan is going through a rapid nutrition transition where shift from underweight to overweight and obesity is occurring. In this paper, we report on the relationship of household socioeconomic position (SEP), community SEP and urbanicity with under- and over-weight categories of BMI among Pakistani women.

**Methods:**

We analyzed data on 4,767 women ages 15-49 years enrolled in a nationally representative Pakistan Demographic Health Survey (PDHS) conducted in 2012-13 that employed a multistage, stratified cluster sampling design. We assessed the association of urbanicity, household and community SEP derived from household assets and utilities, with categories of body mass index (BMI) using multinomial regression analysis where normal weight (BMI 18.6-22.5) was the reference category.

**Results:**

Thirteen percent of women were underweight (BMI <18.5), 15% pre-overweight (BMI: 22.6-24.9), 25% overweight (BMI: 25.0–29.9) and 14% were obese (BMI≥30). Pre-overweight, overweight and obesity among women increased across household wealth quintiles (HWQs) in a graded fashion whereas there was no significant difference in underweight by household wealth. Women in urban areas were more likely to be obese. There was a pronounced increase in adjusted odds ratios (aORs) for overweight/obesity across HWQs within urban areas compared to rural areas. There was a steeper gradient in aORs for obesity from 1st to 5th HWQs in high income communities compared to the middle- and low income communities. In community-level analyses, communities in urban areas were more likely to have higher levels of obesity while in rural areas, especially in Sindh, more communities were more likely to have a higher level of underweight.

**Conclusion:**

A shift to higher overweight and obesity than underweight in Pakistan is associated with high household and community wealth as well as living in urban areas. Clustering of obesity and underweight in distinct communities afford opportunity for tailored intervention programs.

## Introduction

A shift in the nutritional status from under- to over-weight is happening across developing countries, but trends differ according to economic and dietary shifts within countries and regions[1,2]. A substantial proportion of women and children in South Asia (Pakistan, India, and Bangladesh) are under-nourished especially those in lower socioeconomic groups [3–5]. Underweight continues to contribute to poor health outcomes in the developing world, whereas increasing overweight/obesity is resulting in a mounting burden of non-communicable diseases such as diabetes and cardiovascular diseases[4,6,7].

In Pakistan, the overall proportion of underweight among women has decreased from 25% (1993–94 National Health Survey of Pakistan (NHSP)) to 16% (2011 National Nutrition Survey of Pakistan NNSP)) and 13% (2012–13 Pakistan Demographic and Health Survey (PDHS)) over past two decades[8,9]. During the same period, there has also been an increase in the proportion of overweight (body mass index (BMI): ≥ 25) women from 22.5% (1993–94 NHSP) to 34% (2011 NNSP) and 39% (2012–13 PDHS) [8,9]. Regional studies across Pakistan also report a trend of increasing overweight and obesity [10–13]. However, a systemic assessment of reasons for this shift has yet not been performed. Trickledown effect from the overall economic development resulting in increased income and changes in diet and physical activity, as seen elsewhere, have been postulated[14].

During the past few decades, the population size of urban and suburban areas in Pakistan has grown substantially[15]. Living in urban areas has both positive and negative impacts on health. Positive impacts include better access to healthcare, clean water, sanitation, education, social services and better economic opportunities. Negative impacts include exposure to air pollution, noise, crime, low physical activity and poor dietary habits[16,17]. Poor populations living in urban/suburban areas are also exposed to poor living conditions. Studies from China and the Philippines have reported changes in nutritional status with increase in levels of urbanicity [18,19].

Data from the 2012–13 PDHS provide an opportunity to understand the relationship of household and community wealth and urbanicity with nutritional status of women in Pakistan. With the changing micro- and macro-economic environment, the current nutritional programs may not be serving the needs of all women across socioeconomic and urban-rural spectrum. Understanding the factors affecting the nutritional status of women will guide development and implementation of targeted, need-based interventions at household and community levels. The objective of this analysis is to assess the association of household and community socio-economic factors and urbanicity with under- and over-weight categories of BMI among Pakistani women.

## Methods

### Ethics statement

The original survey was reviewed by ICF Institutional Review Board. Analysis reported in this paper were conducted on de-identified data which was considered exempt from the ethics review.

### Setting and survey design

The 2012–13 Pakistan Demographic and Health Survey (PDHS) is a nationally representative household survey conducted in Pakistan. PDHS is a standard demographic and health survey conducted under the umbrella of global Demographic and Health Survey program focusing on women and child health[9]. The households were selected through a stratified two-stage cluster survey design. The first stage involved the selection of 500 primary sampling units(PSU) or clusters based on probability proportional to population size within each stratum. PSUs were census enumeration blocks in urban areas and villages in rural areas. The second stage involved the selection of households. In each PSU, 28 households were selected through systematic sampling resulting in a total selection of 14,000 households across Pakistan. Interviews were conducted with 12,943 households. Federally Administered Northern Areas and restricted military and protected areas were not included in the sample. Smaller provinces (Baluchistan and Khyber Pakhtunkhwa (KPK) and urban areas were over sampled.

### Study population

Ever-married women ages 15-49years who were either a usual resident of the household or a visitor were eligible for interview on women’s questionnaire. Height and weight were measured on a subset of these women recruited from every third selected household, and they formed the study population for the analysis presented in this paper. Women who were pregnant or who had given birth in the two months preceding the survey were excluded from the anthropometric analysis.

### Exposures

The main exposures of interest were household and community socioeconomic position (SEP). A wealth index based on household possessions, utilities and construction was used as an indicator of household SEP. Such indices have shown good validity and reliability in classifying households by their wealth in developing countries and provide a measure of income inequality in health status[20,21]. The household wealth score was obtained by summing scores for each asset with the weight for each asset derived from the principle component analysis[22]. The wealth score is a constructed composite measure and does not have a direct interpretation. Thus, we divided the population into quintiles of the wealth: the bottom quintile represents poorest and 5th quintile represents the richest.

The community wealth score was obtained by aggregating household wealth scores at the community level. A community was defined as the census enumeration block in urban areas and a village in rural areas. Communities were divided into three groups(low, middle and high) based on wealth scores tertiles.

### Covariates

Place of residence (major urban-including large metropolitan cities, small cities and rural areas), province, age, education, occupation, husband’s education, parity and ethnicity were included in the analysis as covariates.

### Outcome assessment

Outcome of interest was BMI(weight(Kg)/height(m)^2^). According to the World Health Organization’s recommendations for Asian populations, we categorized BMI into following groups: <18.5:underweight, 18.5–22.9:normal weight, 23–24.9:pre-overweight or at risk of overweight, 25–27.5:overweight-1, 27.6–29.9:overweight-2, and ≥30(obese)[23].

### Statistical analysis

We used complex survey methods applying sampling weights to account for unequal sampling probabilities and accounting for clustering in our analyses. We computed the proportion of under-weight, normal, pre-overweight, overweight, and obese women.

To assess the relationship between SEP and categories of BMI, we performed multinomial regression analysis where normal weight(BMI:18.6–22.9) was the reference for the underweight category and three categories of overweight. We assessed the interaction of household and community wealth as well as the interaction of household wealth quintiles (HWQ) and place of residence. Place of residence and community wealth were highly correlated; wealthy communities were mainly located in large cities while poor communities were mainly in rural areas.

To assess the variation in under- and over-weight across communities, we fitted a multilevel multinomial regression model for BMI categories using normal weight as reference with communities as a random effect. We used the model based community-level mean probability of being under-weight, overweight and obesity to compute the ratio of probability of underweight to obesity at the community level to assess co-existence of under-weight to obesity. Analyses were performed using SAS 9.3.

## Results

### Participant profile

Overall 13,558 women provided responses in the PDHS. Of these, data for height and weight were available for 4,676 women who were included in the analysis presented in this paper. Mean (SE) age of women was 32.5 (0.18)years [median:31; range:15–49]. The 30-39years age group comprised the largest percentage (33%). About 57% of the women had no formal schooling while 26% completed secondary school or higher. Husbands of 32% of women had no education while 17% had ≥11years of education. Most women(69%) were not working in gainful employment (Table 1). Area of residence was associated with community wealth; 55% of rural communities were poor compared to < 1% in major urban areas and 87% of communities in major urban areas were rich compared to 4% in rural areas(p< 0.001).

**Table 1:** Distribution of BMI by participants’ characteristics, Demographic and Health Survey of Pakistan 2012–13.

		Underweight	Normal	Pre-overweight	Overweight		Obesity
	Overall	<18.5	18.5–22.9	23.0–24.9	25.0–27.5	27.5–29.9	≥30
	N (%)	%(95%CI)	%(95%CI)	%(95%CI)	%(95%CI)	%(95%CI)	%(95%CI)
**Place of residence**							
Major Urban	1017(19)	5(3–7)	23(20–27)	16(13–19)	21(18–24)	14(10–18)	21(19–24)
Urban	1118(15)	10(7–13)	27(22–32)	12(9–15)	15(12–19)	14(12–16)	22(18–26)
Rural	2541(67)	16(14–18)	37(35–39)	15(13–17)	14(12–15)	8(6–9)	11(9–13)
**Province**							
Punjab	1376(58)	13(10–15)	32(29–34)	14(12–16)	15(13–17)	10(8–12)	16(14–19)
Sindh	1024(23)	19(15–22)	39(36–43)	14(12–17)	12(10–15)	7(5–9)	9(7–11)
KPK	925(14)	6(4–8)	26(23–30)	18(15–21)	21(17–24)	13(10–15)	16(12–19)
Baluchistan	632(4)	8(5–11)	35(28–42)	20(15–25)	22(16–28)	8(5–11)	7(3–11)
Gilgit/Baltistan	424(1)	5(2–9)	59(50–67)	22(15–30)	9(5–12)	2(0–5)	3(1–5)
Islamabad (ICT)	295(<1)	7(4–10)	26(22–31)	10(6–14)	14(10–18)	18(13–22)	25(20–29)
**Age (years)**							
15–24	937(22)	19(15–24)	47(43–51)	13(10–16)	10(8–13)	5(3–6)	5(4–7)
25–29	915(19)	15(12–19)	37(33–40)	16(12–19)	15(11–19)	9(7–12)	8(6–11)
30–39	1609(33)	10(8–12)	29(26–33)	15(12–17)	18(15–20)	11(9–13)	18(15–20)
40–49	1215(26)	10(8–12)	23(20–26)	15(13–18)	17(14–19)	13(11–15)	21(18–24)
**Education**							
No education	2616(57)	16(14–18)	37(34–39)	14(13–16)	14(12–15)	8(7–10)	11(9–13)
Primary (1–5 years)	691(17)	12(9–16)	30(26–33)	15(12–18)	16(13–19)	9(6–12)	18(15–22)
Secondary (6–10 years)	826(17)	9(6–12)	28(23–32)	16(12–19)	18(14–22)	11(9–14)	19(15–23)
Higher (≥11 years)	543(9)	5(3–7)	25(21–30)	16(12–20)	19(15–24)	15(11–20)	19(15–24)
**Husband education**							
No education	1447(32)	18(15–20)	38(35–41)	14(12–17)	13(10–16)	8(6–10)	9(7–11)
Primary (1–5 years)	658(17)	15(12–18)	33(28–38)	14(11–18)	14(11–17)	9(7–12)	14(11–18)
Secondary (6–10 years)	1489(34)	11(8–13)	31(28–34)	16(13–18)	16(14–19)	10(8–12)	17(14–20)
Higher (≥11 years)	1067(17)	7(5–9)	27(23–31)	14(10–17)	19(15–23)	14(11–17)	19(15–23)
**Occupation**							
Not working	3586(69)	10(9–12)	31(29–33)	15(13–17)	16(15–18)	11(9–12)	17(15–19)
Professional/technical/managerial/sales/Services	471(11)	11(7–15)	33(28–38)	14(10–18)	18(13–22)	11(8–15)	12(8–16)
Agricultural worker	306(11)	25(19–32)	43(36–50)	13(9–16)	10(6–14)	5(2–8)	4(1–6)
Unskilled/skilled manual	313(9)	22(16–28)	35(29–41)	15(11–20)	11(6–16)	8(4–11)	9(6–13)
**Household wealth quintiles**							
1^st^ quintile (Poorest)	847(19)	24(19–30)	47(43–52)	12(10–14)	8(5–10)	4(2–6)	4(2–6)
2^nd^ quintile	922(19)	16(13–20)	41(37–45)	17(14–20)	13(10–15)	6(4–8)	7(5–9)
3^rd^ quintile	873(19)	13(10–16)	33(28–37)	16(13–20)	16(13–19)	9(6–11)	13(11–16)
4^th^ quintile	968(22)	8(6–11)	27(23–31)	13(10–16)	19(16–22)	13(10–16)	20(17–23)
5^th^ Quintile (Richest)	1066(21)	4(3–6)	20(17–23)	15(12–18)	20(16–24)	16(13–19)	25(22–28)
**Community Wealth**							
Low	1730(37)	21(18–24)	43(40–46)	14(12–16)	11(9–13)	6(4–8)	5(4–6)
Middle	1617(37)	10(8–13)	31(28–34)	16(13–18)	16(14–18)	11(9–12)	16(13–19)
High	1329(26)	6(4–7)	21(18–25)	14(12–16)	20(17–23)	14(11–17)	24(22–27)
**Ethnicity**							
Urdu	447(10)	8(5–10)	25(20–30)	14(10–19)	22(16–29)	14(10–18)	16(12–21)
Punjabi	1057(38)	11(8–13)	28(25–32)	14(12–17)	17(14–19)	11(9–13)	19(16–22)
Sindhi	481(10)	26(20–32)	40(34–45)	12(9–16)	9(6–12)	4(2–6)	8(5–12)
Pushto	991(13)	4(2–6)	28(25–32)	18(15–21)	22(18–25)	13(10–15)	15(11–18)
Balochi	436(6)	23(11–35)	40(34–47)	17(12–21)	12(4–20)	4(1–7)	4(1–7)
Siraiki	481(16)	17(13–21)	46(41–51)	13(10–16)	8(5–10)	7(4–10)	9(6–13)
Hindko/Potowari	243(3)	14(7–22)	26(19–33)	16(9–23)	18(10–26)	11(5–18)	15(9–20)
Shina/Chitrali	400(1)	7(3–10)	54(46–62)	19(15–24)	14(8–20)	3(0–6)	3(0–6)
Others	140(3)	11(5–17)	38(27–48)	17(11–23)	10(5–15)	11(4–18)	14(8–20)
**Watch TV**							
Not at all	1514(32)	18(15–21)	39(36–41)	15(13–18)	14(11–16)	7(5–9)	8(6–10)
Occasionally	905(21)	13(10–16)	33(29–37)	13(10–16)	14(11–18)	12(9–15)	15(12–18)
At least once a week	100(3)	17(8–26)	31(20–43)	11(4–18)	20(10–30)	11(3–18)	10(3–17)
Daily	2155(45)	9(8–11)	29(26–32)	15(13–18)	17(14–19)	11(9–12)	19(16–21)
**Parity**							
0	557(13)	14(11–18)	43(37–49)	13(9–16)	12(9–16)	8(6–11)	9(6–13)
1–3	1865(41)	14(12–17)	34(31–36)	15(13–17)	15(13–17)	10(8–12)	12(10–14)
≥3	2254(46)	11(9–13)	30(27–32)	15(13–17)	17(15–19)	10(9–12)	17(15–20)

A third (33%) of women were normal weight (BMI18.6–22.9), followed by overweight (BMI 25.0–27.5: 15%; BMI 27.5–29.9: 10%), pre-overweight(BMI 23.0–24.9: 15%), obese(BMI ≥30.0: 14%) and underweight(BMI <18.5:13%)(Table 1 and S1 Fig). Overall 54% of the participants were pre-overweight, overweight, or obese and 39% had BMI ≥25.

### Household SEP and underweight

The proportion of underweight decreased from 24% in 1^st^ HWQ to 4% in 5^th^ quintile(Table 1 and S1 Fig). However, after adjusting for other covariates, individuals in the bottom quintile were not more likely to be underweight than those in the 5th quintile in comparison to normal weight individuals (Table 2).

**Table 2:** Multivariable model for association of household socio-economic position and other participants’ characteristics with categories of BMI among women, Demographic and Health Survey of Pakistan 2012–13.

	Adjusted ORs(95% confidence interval)				
	BMI <18.5	BMI 23–24.9	BMI 25.0–27.5	BMI 27.5–29.9	BMI ≥ 30
Covariates	N = 479	N = 756	N = 735	N = 491	N = 646
**Wealth quintile**					
1^st^ quintile (Poorest)	1.0	1.0	1.0	1.0	1.0
2^nd^ quintile	1(0.6–1.5)	1.5(1.1–2.1)	1.5(1–2.2)	1.1(0.6–2)	1.1(0.6–2.2)
3^rd^ quintile	1(0.7–1.7)	1.9(1.3–2.9)	2.4(1.4–4)	2(1–3.7)	2.7(1.4–5.1)
4^th^ quintile	0.9(0.5–1.6)	2.1(1.3–3.3)	3.7(2.3–6)	3.8(1.9–7.3)	4.9(2.4–10.1)
5^th^ Quintile (Richest)	0.7(0.3–1.3)	3.1(1.8–5.3)	4.6(2.6–8.2)	5.2(2.4–11.1)	6.8(3.3–14.2)
**Urbanization**					
Major urban	0.7(0.4–1.1)	1.2(0.7–1.8)	1.2(0.9–1.8)	1.3(0.7–2.5)	1.7(1.1–2.6)
Urban	1(0.7–1.6)	0.8(0.5–1.3)	0.9(0.6–1.3)	1.4(0.9–2)	1.4(0.9–2.2)
Rural	1.0	1.0	1.0	1.0	1.0
**Age (years)**					
15–24	0.9(0.6–1.4)	0.7(0.5–1)	0.6(0.4–1)	0.4(0.3–0.7)	0.6(0.3–0.9)
25–29	1.0	1.0	1.0	1.0	1.0
30–39	0.7(0.5–1.1)	1.2(0.9–1.8)	1.6(1.1–2.3)	1.7(1.1–2.7)	3.1(2–4.8)
40–49	1(0.7–1.5)	1.6(1.1–2.4)	1.9(1.3–3)	2.6(1.7–4)	4.9(3–7.9)
**Husband education (years)**					
No education	1.0	1.0	1.0	1.0	1.0
Primary	1(0.7–1.3)	1.2(0.8–1.8)	1.2(0.8–1.9)	1.4(0.9–2.2)	1.7(1.1–2.7)
Secondary	0.9(0.6–1.2)	1.2(0.9–1.6)	1.1(0.8–1.5)	1(0.7–1.6)	1.5(0.9–2.4)
Higher	0.7(0.4–1.2)	1(0.6–1.5)	1.2(0.8–1.8)	1.3(0.8–2.2)	1.5(0.9–2.4)
**Province**					
Baluchistan	0.5(0.2–0.8)	1.7(1–2.9)	2.2(0.9–5)	1.9(0.9–4.4)	1(0.3–3)
Gilgit	0.3(0.1–0.6)	2(0.9–4.4)	0.8(0.2–2.7)	1.1(0.2–6.5)	1.1(0.3–4.8)
ICT	0.9(0.4–1.7)	0.6(0.3–1)	0.6(0.3–1)	1.1(0.6–1.9)	0.8(0.5–1.3)
KPK	0.8(0.4–1.6)	2.1(1.1–4)	2.5(1.1–5.3)	3.1(1.6–6.4)	2.9(1.5–5.7)
Sindh	0.8(0.6–1.1)	0.9(0.6–1.3)	0.7(0.4–1.3)	0.6(0.4–1)	0.4(0.2–0.7)
Punjab	1.0	1.0	1.0	1.0	1.0
**Occupation**					
Not working	1.0	1.0	1.0	1.0	1.0
Professional/technical/managerial/Sales/Services	0.9(0.6–1.4)	0.9(0.6–1.4)	1.1(0.8–1.5)	1(0.7–1.5)	0.6(0.4–1)
Agricultural—employee	1.3(0.9–2)	0.9(0.5–1.6)	0.9(0.5–1.6)	0.8(0.3–1.7)	0.4(0.2–0.9)
Unskilled/skilled manual	1.5(1–2.3)	1.2(0.8–1.9)	1(0.6–1.7)	1.1(0.6–1.9)	0.8(0.5–1.4)
**Ethnicity**					
Punjabi	1.0	1.0	1.0	1.0	1.0
Urdu	1.4(0.8–2.4)	0.9(0.5–1.5)	1.1(0.6–2.1)	1(0.6–1.6)	0.7(0.4–1.3)
Sindhi	1.9(1.1–3.3)	1(0.6–1.7)	0.9(0.4–2.1)	0.7(0.3–1.4)	1.5(0.7–3.2)
Pashto	0.5(0.2–1)	0.9(0.4–1.7)	0.9(0.4–1.9)	0.7(0.3–1.6)	0.6(0.3–1.3)
Baluchi	1.7(0.9–3)	1.2(0.7–1.9)	0.9(0.3–2.2)	0.5(0.2–1.2)	0.6(0.2–1.8)
Seraiki	0.9(0.6–1.4)	0.7(0.5–1.1)	0.5(0.3–0.7)	0.7(0.4–1.2)	0.7(0.4–1.2)
Hindko	1.9(0.9–4)	0.8(0.4–1.7)	0.7(0.3–1.9)	0.6(0.2–1.6)	0.5(0.2–1.2)
Kashmiri/Gilgiti	0.8(0.4–1.7)	0.5(0.2–1.1)	0.6(0.2–1.8)	0.2(0–0.9)	0.1(0–0.5)
Others	0.9(0.4–1.9)	0.9(0.4–1.8)	0.5(0.2–0.9)	0.8(0.3–2.2)	0.7(0.3–1.5)

. (N = 1569); Reference: BMI = 18.5–22.9

### Household SEP and overweight categories

There was an increase in pre-overweight, overweight and obesity with increasing HWQs (Table 1, S1 Fig and S1 Table). The Multivariable model showed a graded increase in adjusted odds ratios (aOR) for overweight and obesity with increasing HWQs. For those in 5^th^ quintile of HWQs, aORs ranged from 4.6 for overweight-1 to 6.8 for obesity (Table 2).

### Community wealth, residence area and under- and over-weight categories

Women living in major urban areas were more likely to be obese (aOR:1.7; 95%CI: 1.1–2.6). The difference in underweight across place of residence was not significant(Table 2). In the model with interaction of HWQs and place of residence, there was a gradient of increasing aORs with an increase in wealth quintiles for pre-overweight, overweight and obesity within both urban and rural areas. But the difference for obesity was more pronounced between urban and rural areas among those in 5th quintile (aOR: 10.8 vs 7.0) (Table 3 and S2 Table).

**Table 3:** Multivariable models presenting interaction between household socio-economic position and urbanicity, and interaction between household and community socioeconomic position for association with categories of BMI among women, Demographic and Health Survey of Pakistan 2012–13*.

	Adjusted ORs(95% confidence interval)			
Models	BMI <18.5	BMI 23–24.99	BMI 25.0–29.9	BMI ≥ 30
	N = 478	N = 754	N = 1223	N = 646
**Interaction of household wealth quintile(HWQ) and urbanization** ^**†**^				
** Urban**				
HWQ -1^st^ quintile (Poorest)	1.6(0.3–7.7)	3.1(1.1–8.6)	4.9(1.7–14.5)	0.4(0.1–2.3)
HWQ -2^nd^ quintile	0.5(0.3–1)	2(1.1–3.9)	1.1(0.5–2.4)	2.8(0.9–8.7)
HWQ -3^rd^ quintile	1.2(0.6–2.5)	1.7(0.9–3.4)	2.5(1.5–4.4)	3.8(1.6–9)
HWQ -4^th^ quintile	0.7(0.3–1.5)	1.9(1.1–3.2)	4.5(2.9–7.2)	7.3(3.6–14.9)
HWQ -5^th^ Quintile (Richest)	0.6(0.3–1.1)	3.2(2–5.4)	6.2(3.8–10)	10.8(5.4–21.8)
** Rural**				
HWQ -1^st^ quintile (Poorest) (Ref)	1.0	1.0	1.0	1.0
HWQ -2^nd^ quintile	1(0.6–1.6)	1.5(1–2.2)	1.4(1–2.1)	1.1(0.5–2.1)
HWQ -3^rd^ quintile	1(0.6–1.6)	2(1.3–3.1)	2.3(1.5–3.7)	2.7(1.4–5.3)
HWQ -4^th^ quintile	0.9(0.5–1.8)	2.3(1.4–3.9)	3.8(2.4–6.1)	5(2.3–10.6)
HWQ -5^th^ Quintile (Richest)	0.6(0.2–1.6)	3.2(1.5–7)	5.2(2.7–10.2)	7(2.9–17.3)
**Household wealth quintile (HWQ) and community wealth tertile‡**				
** Low community wealth**				
HWQ -1^st^ quintile (Poorest) (Ref)	1.0	1.0	1.0	1.0
HWQ -2^nd^ quintile	0.8(0.5–1.3)	1.5(1–2.3)	1.6(1–2.4)	1(0.4–2.2)
HWQ -3^rd^ quintile	1.3(0.8–2.2)	1.9(1.2–3)	2.3(1.3–4.2)	1.6(0.7–3.9)
HWQ -4^th^ quintile	1.1(0.4–3)	1.4(0.5–4)	4.6(2.1–10)	3.4(1–12.1)
HWQ -5^th^ Quintile (Richest)	NE	NE	6.7(0.9–47.5)	3.6(0.5–23.8)
** Middle community wealth**				
HWQ -1^st^ quintile (Poorest)	0.4(0.1–1.8)	0.8(0.3–2.2)	1.5(0.6–3.8)	0.9(0.2–3.3)
HWQ -2^nd^ quintile	1(0.5–2)	1.4(0.8–2.3)	1.2(0.7–2)	1.5(0.7–3.4)
HWQ -3^rd^ quintile	0.7(0.4–1.2)	1.9(1.1–3.3)	2.3(1.4–3.8)	3.3(1.5–7.1)
HWQ -4^th^ quintile	0.8(0.4–1.7)	2.4(1.4–4.1)	3.8(2.3–6.3)	5.3(2.3–11.9)
HWQ -5^th^ Quintile (Richest)	0.6(0.2–1.6)	2.7(1.4–5.3)	4.5(2.6–7.9)	6.4(2.7–15.2)
** High community wealth**				
HWQ -1^st^ quintile (Poorest)	NE	NE	NE	NE
HWQ -2^nd^ quintile	NE	6.1(2.8–13.3)	1.1(0.1–15.8)	0.3(0–7.5)
HWQ -3^rd^ quintile	2.3(0.7–7.3)	2.1(0.7–6.2)	3.3(1.1–9.7)	4.9(1.6–15.3)
HWQ -4^th^ quintile	0.6(0.2–2)	1.9(0.8–4.1)	4.2(2.2–7.9)	7.2(2.9–18.1)
HWQ -5^th^ Quintile (Richest)	0.6(0.2–1.5)	3.5(1.7–7.3)	5.7(3.1–10.6)	10.3(4.2–25.1)

NE: Not estimable due to small cell count

* Table presents estimates from interaction. Full models presented as S2 Table and S3 Table.

† Model adjusted for age, husband education, province, occupation and ethnicity

‡ Model adjusted for age, place of residence, husband education, province, occupation and ethnicity

In univariable analysis, the risk of underweight decreased with increase in community wealth and, conversely, risk of overweight and obesity increased with an increase in community wealth in a graded fashion (S1 Table). In the multivariable model with an interaction between household and community wealth, there was an increase in aORs for obesity from 1st to 5th quintile across low, middle and high community wealth. For women in the 5th HWQ and low community wealth, the aOR was 3.6(95%CI: 0.5–23.8) compared to 6.4(95%CI: 2.7–15.2) for those in middle community wealth and 10.3(95%CI: 4.2–25.1) for those in high community wealth group(Table 3 and S3 Table). There was no significant association between household wealth and underweight across levels of community SEP(Table 3).

Women with husbands having any education were more likely to be obese compared to those with husbands having no education(aOR: 1.5; 95%CI:1.0–2.3). Compared to women not working in gainful employment, women involved in manual work were more likely to be underweight(aOR:1.5; 95%CI: 1.0–2.3). Women in agricultural work were less likely to be obese(aOR:0.4; 95%CI: 0.2–0.9). Women in managerial/technical and sales jobs were also less likely to be obese(aOR:0.4; 95%CI: 0.2–0.9).

### Co-existence of underweight and obesity at the community level

**Fig 1:**
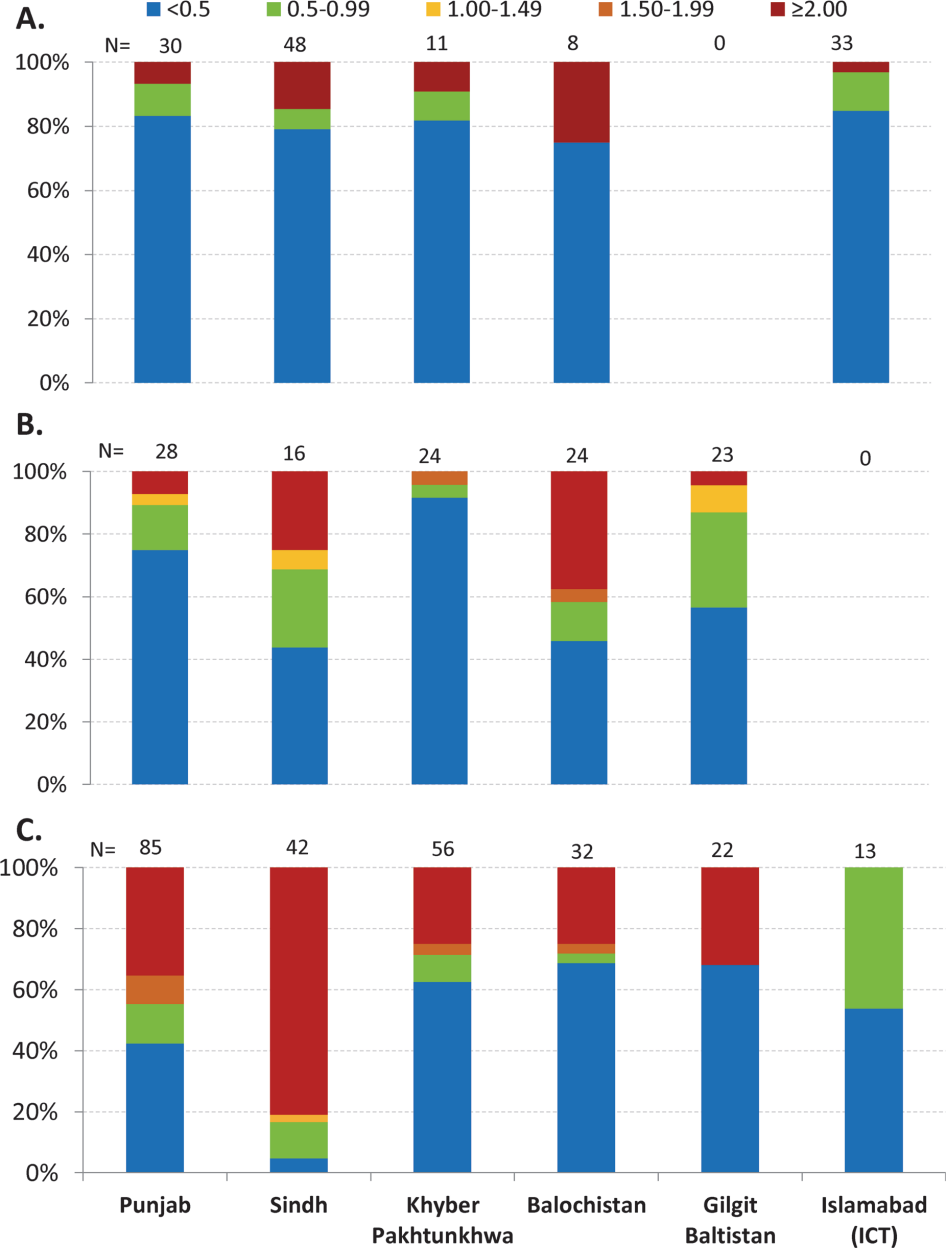
Distribution of ratio of probability of being under-weight to obese at community level by urbanization level, DHS Pakistan 2012–13. A: Major Urban; B: Small City and C: Rural. Ratio of 1 denotes equal percentage of underweight and obesity, below 1 indicates higher obesity and above 1 indicates higher percentage of underweight within a community.

Fig 1 presents the distribution of ratio of community level probability of being underweight to obese by place of residence as major urban, urban and rural across provinces. In most communities in major urban areas, the likelihood of being obese is much higher than being underweight. However, in 14% of communities in major cities of Sindh and 25% communities in major cities of Baluchistan, the likelihood of underweight was higher than obesity. In small cities, the majority of communities had higher obesity compared to underweight with differences across provinces. More than 95% of communities in KPK had higher obesity than underweight compared to < 70% in Sindh and <60% in Baluchistan. In contrast to major urban and small cities, more communities in rural areas had higher underweight compared to obesity especially in Sindh where >80% communities had more underweight than obesity. In Punjab, 45% of communities had more underweight than obesity. These data suggest that communities in urban areas are more likely to have higher levels of obesity while in rural areas especially in Sindh more communities are likely to have a higher level of underweight.

## Discussion

Our analysis of nationally representative sample from Pakistan shows that overweight (BMI ≥25) was three times that of underweight among women. Overweight and obesity increased with an increase in household wealth in a graded fashion whereas there was no significant difference in underweight by wealth. Women from wealthy communities were at further higher risk of overweight and obesity, especially those from richer/richest households compared to other women in their community and poor communities. Women from major urban areas were also at higher risk of overweight and obesity especially those from wealthy households whereas women in rural areas were more likely to be underweight. Exploration of within community risk of underweight and obesity suggested that risk of obesity was higher in most of the communities in urban areas across all provinces whereas in a substantial proportion of communities in rural areas especially in the province of Sindh, the risk of underweight was higher than obesity. These findings have important implications for guiding the nutrition policy and programs in Pakistan in terms of the need for developing programs to address rising overweight and obesity in urban areas and targeting under-weight in high prevalence rural areas.

These findings are consistent with our earlier report from a district of Sindh province where we found that the risk of overweight and obesity increased with increase in household wealth whereas underweight levels did not differ by wealth[13]. However, national level data has provided us an additional insight into nutritional dynamics and transition in Pakistan at national and regional levels. We were able to assess differences in overweight and obesity by geographic region, place of residence and overall community wealth. These findings suggest that overweight and obesity among women increased with increasing household wealth whereas there was no significant difference in underweight by wealth. Women living in urban areas, especially those in large urban metropolitans, are at a higher risk of overweight and obesity. Their risk of overweight and obesity increases further if they live in wealthy communities compared to those living in low income communities. As highlighted above, there has been a decline in underweight among women from 25% in 1993–94 NHSP to 13% in current survey while there has been an increase in overweight from 23% in 1994 to 39% in 2012–13[8]. Socioeconomic gradient and urban rural differences were also noted in 1993–94 NHSP, though increase in overweight and obesity with increasing HWQs is more pronounced in the current data than in the 1994 data. Decline in underweight and increase in overweight since 1994 and high levels of overweight/obesity especially in urban areas suggest rapid nutrition transition in Pakistan requiring policy changes to address the changing nutritional dynamics.

Similar findings on the association of household wealth and increase in overweight and obesity have been reported from India, Bangladesh and Nepal[24–27]. However, the overall prevalence of overweight /obesity in Bangladesh(2007: 10%/1.7%; 2011: 17%/2.9), India(2005–06: 9.8%/2.9%) and Nepal (2011:11.2%/2.2%) is lower than that in Pakistan(25%/14%)[28–30]. A significant decreasing trend in underweight with increasing wealth has been reported from India and Bangladesh, though still a large proportion of women in lowest income quintile are underweight (50% in India and 40% in Bangladesh)[5,24]. We did not find a statistically significant association of household wealth with underweight. There was however, a trend towards reduction in underweight with increase in the HWQs(poorest:24% vs richest:4%; aOR-richest: 0.7, 95%CI:0.3–1.3). The proportion of underweight women in India and Bangladesh is almost twice as that of Pakistan(Pakistan:13% vs. Bangladesh:30%(2007)/ 21%(2011), India:36%(2005–06)[28–30]. Overall, this suggests that Pakistan is in a relatively advanced stage of nutrition transition compared to India or Bangladesh.

It has been suggested that an increase in household wealth is associated with an increase in food consumption and changes in the types of food consumed[31]. High household wealth is also associated with higher intake of meats, fats and fast food, especially in urban areas[32,33]. We previously reported an increase in meat intake frequency with an increase in household wealth in Sindh province of Pakistan[34]. Household wealth is also associated with availability of household amenities and help for household work such as cleaning and food preparation thus affecting energy expenditure and increase in overweight and obesity[35]. Our data also suggest that compared to women who are not employed, women involved in manual labor and agricultural work are at lower risk of obesity.

In this study, women in rich communities were at further higher risk of overweight and obesity especially those from wealthy households. Women in urban areas especially those from wealthy households of major urban areas were also at higher risk of overweight and obesity. Most of the wealthy communities were in major urban areas. Similar findings on the relationship of community wealth and urbanicity with overweight/obesity have been reported from India and Bangladesh and many other developing countries[5,24,25,36]. Many aspects of these cities promote overweight and obesity including availability of fast-food and other restaurants and increasing norms of eating out at restaurants, mechanized transportation, sedentary jobs and low physical activity[37–39]. A study from India reported a higher fast-food consumption among people living in high income neighborhoods, but such data are not available for Pakistan[40]. Furthermore, lack of parks, walk-able spaces, pollution, and actual and perceived safety, violence/political instability and cultural norms may constraint physical activity among women[41–43]. Data on aspects of neighborhood characteristics that influence overweight and obesity were not available in this study. Further work is needed to characterize aspects of neighborhoods including food and physical activity environment that influence overweight and obesity in Pakistan. Such data from developed countries may not be applicable to situations in developing countries. For example, in developed countries, fast-food density is lower in high income neighborhoods and they provide safe environment for physical activity while the situation may be different in developing countries[37,39].

Exploration of within community risk of underweight and obesity also suggests that risk of obesity is higher in most of the communities in urban areas across all provinces whereas in a substantial number of rural communities, especially in Sindh province, the risk of underweight is higher than obesity. These data suggest that the community level risk of underweight and obesity could be used to tailor nutritional programs according to the need of an area. Areas with a higher probability of overweight/obesity require programs aiming at reducing overweight and obesity by promoting healthy eating and physical activity while areas with a high level of underweight programs could focus on measures to reduce underweight.

Provincial differences in the level of under- and over-weight may be related to poor food access in Sindh province and restriction on physical activity related to the conservative culture in KPK. Investigation into household and macro-level food consumption and access, and under- and over-nutrition in Pakistan is needed. The high level of overweight and obesity in KPK requires further investigation into diet and physical activity determinants including cultural norms restricting women’s mobility[44–46].

This study included ever married women of reproductive age group(15-49years), thus data may not be generalizable to all women in Pakistan. Furthermore, data were collected on a subset of women with smaller sample size leading to instability in analyses involving interactions and limiting our ability in fully exploring differences across urban/rural spectrum. Furthermore, data on characteristics of communities could also help in program planning in identifying community characteristics related to underweight or overweight. Similarly, data to characterize urbanicity in more refined categories would be useful in developing a better understanding of nutrition transition and differences across the rural-urban continuum. Data on dietary intake were not available; hence we were not able to adjust for dietary pattern which may have contributed to potential residual confounding, though effect of wealth may be mediated through diet. In our previous study, the association of household wealth with under- and over nutrition remained strong, even after adjusting for dietary intake [13]. Finally, associations presented from a cross-sectional survey should be interpreted with caution as the temporal relationship between exposure (socio-economic positions) and outcome (overweight/obesity) cannot be ascertained.

In summary, these data suggest that overall level of overweight and obesity has increased substantially among Pakistani women of reproductive age group especially in urban areas. Underweight is still a problem among relatively smaller group of women mainly in rural area especially in the province of Sindh. This calls for re-assessing national policy on nutritional disorders in Pakistan. Current programs focus on under-weight with policies mainly aimed at providing cash incentives and provision of “nutritious foods”[47,48]. Given there is dual burden of under- and over-weight shifting more towards overweight and obesity(BMI≥23: 54%), policy and programs need to re-evaluate their focus on tackling nutritional problems. This could be achieved through comprehensive policy for full spectrum of nutrition with a focus on underweight related interventions/programs in areas where prevalence and risk of underweight is high, and implementing programs for tackling overweight and obesity in areas(urban areas) where it is becoming a major problem. Furthermore, the under nourished segment of the population will also benefit from policies related to healthy diets and the regulation of empty calories/junk food such as sugar-sweetened beverages in the short and long run as seen elsewhere[1,49]. Similarly, an important challenge for development partners in Pakistan and other developing countries is to develop a long-term vision and strategy for achieving health and wellbeing by addressing food insecurity and hunger issues without adding to the burden of overweight and obesity and addressing both of these issues at the same time. As we have learned from middle to high income countries, eventually the burden of under-nutrition in the poor shifts to over-nutrition some of which may be related to policies and programs aimed at reducing under-nutrition[50–52].

## Supporting Information

S1 Fig Distribution of BMI among women by wealth quintiles. (DOCX)

S1 Table Univariable models for association socio-economic position and other characteristics with categories of BMI. (DOCX)

S2 Table Multivariable model presenting interaction of household socio-economic position and urbanicity. (DOCX)

S3 Table Multivariable model presenting interaction of household socio-economic position and community socio-economic position. (DOCX)
