# Supplementary material for: Association of Household and Community Socioeconomic Position and Urbanicity with Underweight and Overweight among Women in Pakistan
Source: PLoS One. 2015 Apr 2;10(4):e0122314. doi: 10.1371/journal.pone.0122314 (PMC4383475; doi:10.1371/journal.pone.0122314)
Supplement: S1 Fig — (DOCX) [file pone.0122314.s001.docx]

**S1 Fig. Distribution of BMI among women by wealth quintiles**
