# Supplementary material for: Association of Household and Community Socioeconomic Position and Urbanicity with Underweight and Overweight among Women in Pakistan
Source: PLoS One. 2015 Apr 2;10(4):e0122314. doi: 10.1371/journal.pone.0122314 (PMC4383475; doi:10.1371/journal.pone.0122314)
Supplement: S1 Table — (DOCX) [file pone.0122314.s002.docx]

**S1 Table. Univariable models for association socio-economic position and other characteristics with categories of BMI**

(N=1569); Reference: BMI= 18.5-22.9

|  | **ORs (95% confidence interval)** | | | | |
| --- | --- | --- | --- | --- | --- |
|  | **BMI <18.5** | **BMI 23 -24.9** | **BMI 25.0 - 27.5** | **BMI 27.5 -29.9** | **BMI ≥ 30** |
| **Covariates** | N=479 | N=756 | N=735 | N=491 | N=646 |
| **Wealth quintile** |  |  |  |  |  |
| 1^st^ quintile (Poorest) | 1.0 | 1.0 | 1.0 | 1.0 | 1.0 |
| 2^nd^ quintile | 0.8(0.5 -1.2) | 1.6(1.2 -2.2) | 1.8(1.2 -2.8) | 1.7(1 -2.9) | 1.9(1 -3.5) |
| 3^rd^ quintile | 0.8(0.5 -1.2) | 1.9(1.4 -2.8) | 2.9(1.9 -4.5) | 2.9(1.6 -5.3) | 4.8(2.7 -8.5) |
| 4^th^ quintile | 0.6(0.4 -1) | 2(1.4 -2.8) | 4.2(2.8 -6.4) | 5.3(3 -9.3) | 8.8(4.9 -15.7) |
| 5^th^ Quintile ( Richest) | 0.4(0.3 -0.6) | 3(2.1 -4.2) | 6.1(3.9 -9.4) | 8.6(4.9 -15) | 14.7(8.4 -25.6) |
| **Urbanization** |  |  |  |  |  |
| Major urban | 0.5(0.4 -0.7) | 1.7(1.2 -2.3) | 2.4(1.8 -3.2) | 2.8(1.8 -4.3) | 3.2(2.4 -4.2) |
| Urban | 0.9(0.6 -1.3) | 1.1(0.7 -1.7) | 1.6(1.1 -2.3) | 2.5(1.8 -3.5) | 2.9(2 -4.3) |
| Rural | 1.0 | 1.0 | 1.0 | 1.0 | 1.0 |
| **Age (years)** |  |  |  |  |  |
| 15-24 | 1(0.7 -1.5) | 0.7(0.5 -0.9) | 0.5(0.3 -0.9) | 0.4(0.2 -0.6) | 0.5(0.3 -0.8) |
| 25-29 | 1.0 | 1.0 | 1.0 | 1.0 | 1.0 |
| 30-39 | 0.8(0.6 -1.1) | 1.2(0.8 -1.7) | 1.5(1 -2.1) | 1.5(1 -2.2) | 2.6(1.8 -3.8) |
| 40-49 | 1.1(0.7 -1.6) | 1.5(1.1 -2.2) | 1.7(1.1 -2.6) | 2.2(1.5 -3.3) | 3.9(2.6 -5.9) |
| **Education** |  |  |  |  |  |
| No education | 1.0 | 1.0 | 1.0 | 1.0 | 1.0 |
| Primary (1-5 years) | 1(0.7 -1.4) | 1.3(0.9 -1.8) | 1.5(1.1 -2) | 1.4(0.9 -2) | 2.1(1.5 -2.9) |
| Secondary (6-10 years) | 0.8(0.5 -1.1) | 1.5(1.1 -2) | 1.8(1.2 -2.5) | 1.8(1.3 -2.6) | 2.3(1.5 -3.4) |
| Higher (≥11 years) | 0.5(0.3 -0.8) | 1.6(1.1 -2.4) | 2.1(1.4 -3) | 2.7(1.8 -4) | 2.6(1.7 -3.8) |
| **Husband's education** |  |  |  |  |  |
| No education | 1.0 | 1.0 | 1.0 | 1.0 | 1.0 |
| Primary (1-5 years) | 1(0.7 -1.3) | 1.1(0.8 -1.7) | 1.2(0.8 -1.9) | 1.4(0.9 -2.1) | 1.8(1.2 -2.7) |
| Secondary (6-10 years) | 0.8(0.5 -1) | 1.4(1.1 -1.8) | 1.6(1.1 -2.1) | 1.5(1.1 -2.2) | 2.4(1.6 -3.5) |
| Higher (≥11 years) | 0.6(0.4 -0.9) | 1.3(0.9 -1.9) | 2.1(1.4 -3) | 2.5(1.7 -3.8) | 3(2 -4.5) |
| **Province** |  |  |  |  |  |
| Baluchistan | 0.6(0.4 -0.9) | 1.3(0.9 -1.9) | 1.3(0.8 -2.1) | 0.7(0.4 -1.3) | 0.4(0.2 -0.8) |
| Gilgit | 0.2(0.1 -0.5) | 0.9(0.5 -1.4) | 0.3(0.2 -0.5) | 0.1(0.1 -0.3) | 0.1(0 -0.2) |
| ICT | 0.6(0.4 -1.1) | 0.9(0.5 -1.5) | 1.1(0.8 -1.7) | 2.1(1.3 -3.2) | 1.8(1.3 -2.5) |
| KPK | 0.6(0.4 -0.8) | 1.6(1.2 -2.1) | 1.7(1.2 -2.2) | 1.5(1.1 -2.1) | 1.2(0.8 -1.6) |
| Sindh | 1.2(0.9 -1.6) | 0.8(0.6 -1.1) | 0.7(0.5 -0.9) | 0.6(0.4 -0.8) | 0.4(0.3 -0.6) |
| Punjab | 1.0 | 1.0 | 1.0 | 1.0 | 1.0 |
| **Occupation** |  |  |  |  |  |
| Not working | 1.0 | 1.0 | 1.0 | 1.0 | 1.0 |
| Professional/technical  l/managerial/Sales/Services | 1(0.7 -1.6) | 0.9(0.6 -1.3) | 1(0.7 -1.4) | 1(0.7 -1.5) | 0.7(0.5 -1.1) |
| Agricultural - employee | 1.8(1.2 -2.7) | 0.6(0.4 -0.9) | 0.4(0.3 -0.7) | 0.3(0.2 -0.7) | 0.2(0.1 -0.3) |
| Unskilled/skilled manual | 1.9(1.3 -2.9) | 0.9(0.6 -1.3) | 0.6(0.4 -1) | 0.7(0.4 -1.1) | 0.5(0.3 -0.8) |
| **Ethnicity** |  |  |  |  |  |
|  | 1.0 | 1.0 | 1.0 | 1.0 | 1.0 |
| Urdu | 0.8(0.5 -1.4) | 1.2(0.8 -1.8) | 1.5(1 -2.4) | 1.5(1 -2.2) | 1(0.6 -1.6) |
| Sindhi | 1.7(1.1 -2.7) | 0.6(0.4 -0.9) | 0.4(0.2 -0.6) | 0.3(0.1 -0.5) | 0.3(0.2 -0.5) |
| Pashto | 0.4(0.2 -0.6) | 1.3(0.9 -1.8) | 1.3(1 -1.8) | 1.1(0.8 -1.7) | 0.8(0.6 -1.2) |
| Baluchi | 1.5(0.8 -2.6) | 0.8(0.5 -1.3) | 0.5(0.2 -1.1) | 0.2(0.1 -0.6) | 0.2(0.1 -0.3) |
| Seraiki | 1(0.7 -1.5) | 0.6(0.4 -0.8) | 0.3(0.2 -0.4) | 0.4(0.2 -0.6) | 0.3(0.2 -0.5) |
| Hindko | 0.3(0.2 -0.6) | 0.7(0.5 -1) | 0.4(0.2 -0.8) | 0.2(0.1 -0.4) | 0.1(0 -0.2) |
| Kashmiri/Gilgiti | 1.5(0.8 -2.7) | 1.2(0.7 -2.3) | 1.2(0.6 -2.2) | 1.1(0.6 -2.2) | 0.9(0.5 -1.6) |
| Kashmiri/Gilgiti | 0.8(0.4 -1.6) | 0.9(0.5 -1.7) | 0.4(0.2 -0.8) | 0.7(0.3 -1.8) | 0.6(0.3 -1.1) |
| **Community wealth tertiles** |  |  |  |  |  |
| 1st (Low) | 1.0 | 1.0 | 1.0 | 1.0 | 1.0 |
| 2^nd^ (Middle) | 0.7(0.5 -0.9) | 1.5(1.1 -1.9) | 2(1.5 -2.7) | 2.4(1.7 -3.5) | 4.4(3.1 -6.2) |
| 3^rd^ (High) | 0.6(0.4 -0.8) | 2(1.4 -2.7) | 3.7(2.7 -5.1) | 4.8(3.1 -7.4) | 9.7(6.9 -13.6) |
| **Parity** |  |  |  |  |  |
| 0 | 1.0 | 1.0 | 1.0 | 1.0 | 1.0 |
| 1-3 | 1.3(0.9 -1.9) | 1.5(1.1 -2.2) | 1.6(1 -2.4) | 1.5(1 -2.4) | 1.7(1.1 -2.5) |
| >3 | 1.2(0.8 -1.7) | 1.7(1.2 -2.6) | 2(1.3 -3) | 1.8(1.1 -2.7) | 2.7(1.8 -4) |
