# Supplementary material for: Association of Household and Community Socioeconomic Position and Urbanicity with Underweight and Overweight among Women in Pakistan
Source: PLoS One. 2015 Apr 2;10(4):e0122314. doi: 10.1371/journal.pone.0122314 (PMC4383475; doi:10.1371/journal.pone.0122314)
Supplement: S3 Table — (DOCX) [file pone.0122314.s004.docx]

**S3 Table. Multivariable model presenting interaction of household socio-economic position and community socio-economic position**

(N=1569); Reference: BMI= 18.5-22.9

|  | **Adjusted ORs(95% confidence interval)** | | | |
| --- | --- | --- | --- | --- |
|  | **BMI <18.5** | **BMI 23 -24.99** | **BMI 25.0 -29.9** | **BMI ≥ 30** |
| **Covariates** | N=478 | N=754 | N=1223 | N =646 |
| **Household wealth quintile (HWQ) and Community wealth tertile** |  |  |  |  |
| **Low community wealth** |  |  |  |  |
| HWQ -1^st^ quintile (Poorest) (Ref) | 1.0 | 1.0 | 1.0 | 1.0 |
| HWQ -2^nd^ quintile | 0.8(0.5 -1.3) | 1.5(1 -2.3) | 1.6(1 -2.4) | 1(0.4 -2.2) |
| HWQ -3^rd^ quintile | 1.3(0.8 -2.2) | 1.9(1.2 -3) | 2.3(1.3 -4.2) | 1.6(0.7 -3.9) |
| HWQ -4^th^ quintile | 1.1(0.4 -3) | 1.4(0.5 -4) | 4.6(2.1 -10) | 3.4(1 -12.1) |
| HWQ -5^th^ Quintile ( Richest) | NE | NE | 6.7(0.9 -47.5) | 3.6(0.5 -23.8) |
| **Middle community wealth** |  |  |  |  |
| HWQ -1^st^ quintile (Poorest) | 0.4(0.1 -1.8) | 0.8(0.3 -2.2) | 1.5(0.6 -3.8) | 0.9(0.2 -3.3) |
| HWQ -2^nd^ quintile | 1(0.5 -2) | 1.4(0.8 -2.3) | 1.2(0.7 -2) | 1.5(0.7 -3.4) |
| HWQ -3^rd^ quintile | 0.7(0.4 -1.2) | 1.9(1.1 -3.3) | 2.3(1.4 -3.8) | 3.3(1.5 -7.1) |
| HWQ -4^th^ quintile | 0.8(0.4 -1.7) | 2.4(1.4 -4.1) | 3.8(2.3 -6.3) | 5.3(2.3 -11.9) |
| HWQ -5^th^ Quintile ( Richest) | 0.6(0.2 -1.6) | 2.7(1.4 -5.3) | 4.5(2.6 -7.9) | 6.4(2.7 -15.2) |
| **High community wealth** |  |  |  |  |
| HWQ -1^st^ quintile (Poorest) | NE | NE | NE | NE |
| HWQ -2^nd^ quintile | NE | 6.1(2.8 -13.3) | 1.1(0.1 -15.8) | 0.3(0 -7.5) |
| HWQ -3^rd^ quintile | 2.3(0.7 -7.3) | 2.1(0.7 -6.2) | 3.3(1.1 -9.7) | 4.9(1.6 -15.3) |
| HWQ -4^th^ quintile | 0.6(0.2 -2) | 1.9(0.8 -4.1) | 4.2(2.2 -7.9) | 7.2(2.9 -18.1) |
| HWQ -5^th^ Quintile ( Richest) | 0.6(0.2 -1.5) | 3.5(1.7 -7.3) | 5.7(3.1 -10.6) | 10.3(4.2 -25.1) |
| **Urbanization** |  |  |  |  |
| Major urban | 0.7(0.3 -1.4) | 1.1(0.6 -1.9) | 1.2(0.8 -1.8) | 1.3(0.8 -2.1) |
| Urban | 1(0.7 -1.7) | 0.8(0.5 -1.3) | 1.1(0.8 -1.5) | 1.2(0.8 -1.9) |
| Rural | 1.0 | 1.0 | 1.0 | 1.0 |
| **Age (years)** |  |  |  |  |
| 15-24 | 0.8(0.5 -1.4) | 0.7(0.4 -1.2) | 0.3(0.2 -0.7) | 0(0 -0.1) |
| 25-29 | 1.0 | 1.0 | 1.0 | 1.0 |
| 30-39 | 0.8(0.6 -1.1) | 1.5(1.1 -2) | 2(1.6 -2.6) | 3.5(2.5 -4.9) |
| 40-49 | 1(0.7 -1.5) | 1.9(1.4 -2.7) | 2.7(2 -3.5) | 5.5(3.8 -8) |
| **Husband's education** |  |  |  |  |
| No education | 1.0 | 1.0 | 1.0 | 1.0 |
| Primary (1-5 years) | 0.9(0.7 -1.3) | 1.2(0.8 -1.8) | 1.3(0.9 -1.8) | 1.7(1.1 -2.8) |
| Secondary (6-10 years) | 0.8(0.6 -1.2) | 1.2(0.9 -1.6) | 1.1(0.8 -1.4) | 1.4(0.9 -2.3) |
| Higher (≥11 years) | 0.7(0.4 -1.2) | 1(0.6 -1.5) | 1.2(0.9 -1.8) | 1.5(0.9 -2.4) |
| **Province** |  |  |  |  |
| Baluchistan | 0.4(0.2 -0.8) | 1.8(1 -3.1) | 2.1(1 -4.4) | 1.1(0.4 -3.3) |
| Gilgit | 0.3(0.1 -0.6) | 1.9(0.8 -4.3) | 0.9(0.3 -2.6) | 1.2(0.3 -5.4) |
| ICT | 0.9(0.4 -1.7) | 0.5(0.3 -1) | 0.8(0.5 -1.2) | 0.8(0.5 -1.2) |
| KPK | 0.8(0.4 -1.6) | 2.1(1.1 -4.1) | 2.7(1.4 -5.1) | 2.9(1.5 -5.6) |
| Sindh | 0.8(0.6 -1.1) | 0.9(0.6 -1.3) | 0.7(0.4 -1.1) | 0.4(0.2 -0.7) |
| Punjab | 1.0 | 1.0 | 1.0 | 1.0 |
| **Occupation** |  |  |  |  |
| Not working | 1.0 | 1.0 | 1.0 | 1.0 |
| Professional/technical/managerial/Sales/Services | 0.9(0.6 -1.4) | 0.9(0.6 -1.4) | 1(0.8 -1.4) | 0.6(0.4 -1) |
| Agricultural - employee | 1.3(0.8 -1.9) | 0.9(0.5 -1.6) | 0.9(0.5 -1.5) | 0.4(0.2 -1) |
| Unskilled/skilled manual | 1.4(0.9 -2.2) | 1.3(0.8 -2) | 1(0.7 -1.6) | 0.8(0.5 -1.5) |
| **Ethnicity** |  |  |  |  |
| Punjabi | 1.0 | 1.0 | 1.0 | 1.0 |
| Urdu | 1.4(0.8 -2.5) | 0.9(0.5 -1.5) | 1(0.6 -1.7) | 0.7(0.4 -1.3) |
| Sindhi | 1.8(1 -3.1) | 1(0.6 -1.8) | 0.8(0.4 -1.6) | 1.7(0.8 -3.7) |
| Pashto | 0.5(0.2 -0.9) | 0.8(0.4 -1.7) | 0.8(0.4 -1.6) | 0.7(0.3 -1.5) |
| Baluchi | 1.5(0.9 -2.7) | 1.2(0.7 -2) | 0.7(0.3 -1.7) | 0.6(0.2 -2) |
| Seraiki | 0.9(0.6 -1.4) | 0.8(0.5 -1.2) | 0.5(0.4 -0.8) | 0.8(0.5 -1.3) |
| Hindko/Potowari | 1.8(0.8 -4) | 0.8(0.4 -1.7) | 0.7(0.3 -1.4) | 0.5(0.2 -1.2) |
| Shina/Chitrali | 0.8(0.4 -1.6) | 0.6(0.3 -1.2) | 0.4(0.1 -1.2) | 0.1(0 -0.7) |
| Others | 0.8(0.4 -1.8) | 0.9(0.4 -1.8) | 0.6(0.3 -1.3) | 0.8(0.4 -1.6) |
